# The application of artificial intelligence in forensic pathology: a systematic literature review

**DOI:** 10.3389/fmed.2025.1583743

**Published:** 2025-07-24

**Authors:** Francesco Orsini, Andrea Cioffi, Luigi Cipolloni, Roberta Bibbò, Angelo Montana, Stefania De Simone, Camilla Cecannecchia

**Affiliations:** ^1^Department of Clinical and Experimental Medicine, Section of Legal Medicine, University of Foggia, Foggia, Italy; ^2^Department of Biomedical Sciences and Public Health, University Politecnica delle Marche, Ancona, Italy

**Keywords:** artificial intelligence, forensic medicine, forensic pathology, bioethics, AI

## Abstract

**Introduction:**

Recent advancements in Artificial Intelligence have shown immense potential across various domains of healthcare, including forensic pathology. This systematic review aims to evaluate the latest innovations brought by Artificial Intelligence in forensic pathology and provide insights into future directions in this evolving field.

**Methods:**

A systematic literature search was conducted using databases for papers published from 1990 to 2025. The search strategy combined terms related to artificial intelligence, forensic odontology, forensic psychiatry and forensic medicine/pathology. Following PRISMA guidelines, 65 articles were initially identified, of which 18 met the inclusion criteria after applying exclusion criteria.

**Results:**

Artificial Intelligence applications demonstrated significant success across multiple forensic domains. In post-mortem analysis, deep learning achieved 70–94% accuracy in neurological forensics. Wound analysis systems showed high accuracy rates (87.99–98%) in gunshot wound classification. Artificial Intelligence-enhanced diatom testing for drowning cases achieved precision scores of 0.9 and recall scores of 0.95. Microbiome analysis applications reached accuracy rates up to 90% for individual identification and geographical origin determination. AI shows promise in forensic age estimation, psychiatric risk assessment, and insanity evaluations.

**Discussion:**

While Artificial Intelligence shows promise as a supportive tool in forensic pathology, several limitations persist, including small sample sizes and variable performance across different applications. Artificial Intelligence serves best as an enhancement rather than a replacement for human expertise. Future development should focus on larger datasets, specialized systems for different forensic applications, and improved interpretability of Artificial Intelligence decisions for legal contexts. The integration of Artificial Intelligence in forensic pathology represents a significant advancement, requiring careful balance between technological innovation and human expertise for optimal implementation.

## 1 Introduction

Advancements in technology have significantly impacted the field of medicine, offering unprecedented tools and methodologies for diagnosing and treating a wide array of diseases. Indeed, biomedical progress has even enabled the prolongation of life for patients suffering from incurable diseases (1, 2). One of the most rapidly evolving areas of technological innovation is artificial intelligence (AI), which has shown immense potential across various domains of healthcare.

AI is a term coined by the computer scientist John McCarthy in 1956, but the British mathematician Alan Turing had already discussed the possibility of machines simulating human thought in 1950. AI has since evolved significantly, enabling the performance of complex tasks quickly and the processing of new information based on previously evaluated data. In recent years, AI has become a central technology, integrated into many aspects of daily life such as personal assistants, internet applications, and business solutions. AI is also increasingly used in various fields of medicine, including forensic pathology (FP) (3, 4). FP aids in the administration of justice and benefits society by supporting crime detection, investigation, and providing expert witnesses in court. This specialization merges medicine and law, following medical and technological advancements from the past to modern times (5). As a result, FP plays a pivotal role in ensuring that the judicial system functions efficiently and accurately, leveraging modern technology to solve crimes and provide critical insights in legal cases.

The intricate nature of modern criminal investigations often necessitates a seamless integration of knowledge and techniques from various forensic disciplines. FP, with its focus on determining the cause and manner of death, lays the foundational understanding of injuries and their origins. This is intrinsically linked with legal medicine, which bridges medical science with legal principles, ensuring that medical findings are accurately interpreted and applied within the legal framework. Furthermore, the psychological aspects of human behavior, motivations, and mental states, pivotal for profiling, understanding criminal intent, and assessing competency, fall within the purview of forensic psychiatry. In the context of modern investigative techniques, the collaboration among these fields is paramount. For instance, a forensic pathologist's findings from an autopsy might suggest a specific type of injury requiring further analysis from a legal medicine perspective to determine its legal implications. Concurrently, forensic psychiatrists may contribute by evaluating the mental state of suspects or victims, offering insights into complex cases involving psychological factors. AI's advancements are poised to further strengthen these interdisciplinary connections. For example, AI-powered image analysis tools could assist forensic pathologists in identifying subtle patterns of injury, while natural language processing (NLP) might aid legal medicine practitioners in sifting through vast amounts of legal documentation to extract relevant precedents. Similarly, AI could help forensic psychiatrists analyze behavioral patterns or linguistic cues in digital communications, providing a more comprehensive understanding of an individual's psychological state. This collaborative approach, augmented by technological innovations, enhances the overall precision and efficacy of investigations, ultimately contributing to a more just and informed legal process.

Initially, to better understand the implications of AI in FP, it is essential to elucidate the meanings of terms such as machine learning (ML), deep learning (DL), NLP, artificial neural networks (ANNs), and convolutional neural networks (CNNs).

ML is a statistical technique for training models with data to predict outcomes, commonly used in precision medicine. This often requires supervised learning with known outcome variables. DL, a type of neural network with multiple layers, learns data representations and extracts complex features for tasks like computer vision and natural language processing, aiding disease detection in medical imaging. NLP uses computational techniques to analyze text, aiming for human-like language processing to structure healthcare information and extract details for decision-making. ANNs, a class of AI algorithms, address various learning aspects. CNNs are neural networks with learnable weights, specifically designed for structured inputs like images, by sharing weights across locations and local neuron responses (6).

In recent years, countless research endeavors have been carried out in the forensic domain focusing on AI and the potential for this technology to address and surpass the constraints of conventional FP methods. Consequently, AI has the capability to enhance the efficiency and accuracy of human specialists.

This study aims to discuss the latest innovations brought by AI in FP and provide insights into future reflections on this still largely unexplored field. By examining recent advancements, the intention is to highlight the potential of AI technologies and stimulate further research and discussion on the future directions of FP. Indeed, the key objectives should be evaluating AI's effectiveness across different forensic applications, from post-mortem analysis to wound classification, assessing accuracy rates and limitations of AI systems in forensic contexts, identifying areas where AI can most effectively support forensic pathologists' work and examining emerging technologies and methodologies that combine AI with traditional forensic techniques. This comprehensive assessment would help forensic pathologists understand both the potential and limitations of AI integration in their practice.

## 2 Methods

We conducted a systematic literature search, critically appraised the collected studies, and performed an electronic search of Medline/PubMed, Cochrane Library and SCOPUS for papers published from 1990 to 2025. The detailed search string strategically combined Medical Subject Headings terms and keywords to maximize both sensitivity and specificity, specifically {[(artificial intelligence) AND (ai)] AND (forensic medicine)} AND (forensic pathology) AND (legal medicine) AND (forensic odontology) AND (forensic psychiatry).

This approach aimed at capturing all relevant research at the critical intersection of AI and various forensic disciplines.

This systematic search initially yelded a total of 65 articles. We then rigorously evaluated each study methodologically according to the PRISMA standards (7), including assessment of bias.

**Figure 1:**
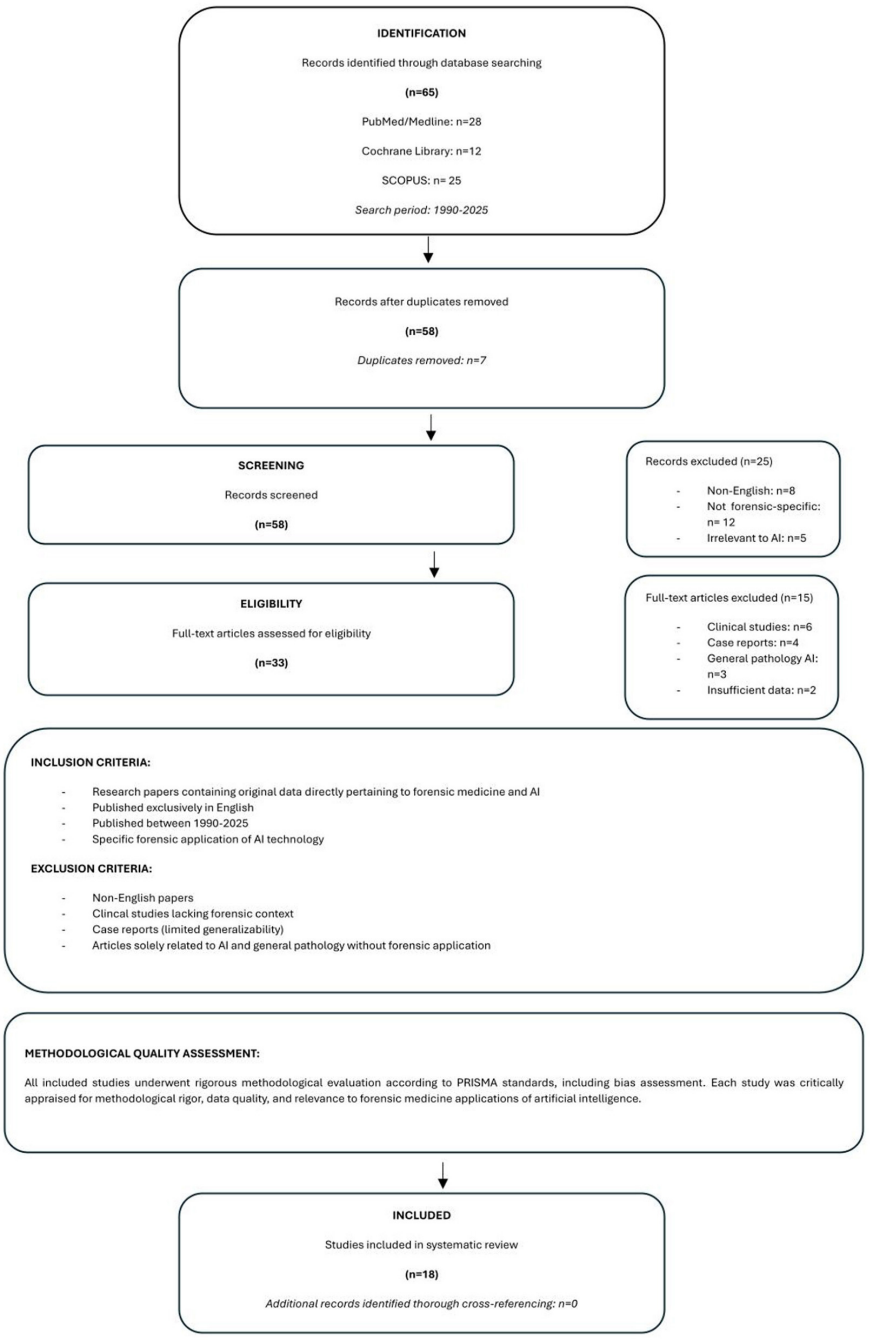
Search strategy following PRISMA criteria.

Inclusion criteria were strictly defined: we sought research papers containing original data directly pertaining to forensic medicine and AI, published exclusively in English. This stringent section ensured the direct relevance and consistency of the reviewed literature. Conversely, several exclusion criteria were applied to enhance the quality and focus of the review. We excluded non-English papers due to the potential for significant translation challenges and inherent risks of misinterpretation, which would compromise the accuracy and comparability of findings. Similarly, clinical studies were omitted as they often lack the specific forensic context crucial to this review's objectives. Case reports were also excluded because of their limited generalizability and higher susceptibility to report bias. Finally, articles solely related to AI and pathology in general, without a specific forensic application, were also excluded to maintain the review's precisely defined scope. Figure 1 visually illustrates the PRISMA flow diagram detailing our selection process. Following this meticulous analysis of the pertinent articles, 18 studies were ultimately included in this systematic review, while 47 were excluded. To ensure comprehensive coverage of the field, the bibliographies of all identified documents were thoroughly reviewed and cross-referenced to uncover any additional relevant literature.

The available quality assessment frameworks for the selected studies were found to be of limited applicability to the specific subject under investigation; therefore, the authors chose not to employ them in this review.

## 3 Results

This systematic review has identified various situations where AI has improved the ability to identify causes, distinguish different types of traumas, and assist in histopathological investigations. Recent advances in AI-assisted post-mortem analysis, forensic odontology, and forensic psychiatry have revolutionized several key areas of forensic investigation (Table 1).

**Table 1:** Characteristics of the analyzed papers.

**Authors**	**Sample size**	**AI technique**	**Forensic application**	**Methodology**	**Main findings**	**Limitations**
Vilmont et al. (8)	86 autopsies (54 males)	Deep learning algorithm	Post-mortem kidney histopathology	Use of a DL method to quickly count glomerular number and measure glomerular density and volume in post-mortem kidney samples. Autopsies (2009–2015) of adults without kidney disease, diabetes, or hypertension were analyzed (86 samples, 54 males).	No significant sex differences in GD or glomerular size. GD inversely correlated with age, kidney weight, and glomerular area, while glomerular area and volume increased with age.	Limited to post-mortem samples; findings not generalizable to populations with kidney diseases or other medical conditions.
Garland et al. (9)	50 PMCT cases (25 injuries, 25 controls)	Convolutional Neural Networks	Post-mortem head injury detection	Investigation of CNNs for Post-Mortem PMCT head imaging. PMCT images from 25 fatal head injury cases and 25 controls were used for training and testing.	Accuracy ranged from 70% to 92.5%. Potential applications include screening tools or computer-assisted diagnostics.	Difficulty recognizing subarachnoid hemorrhage and distinguishing congested vessels from head injuries.
Zirn et al. (10)	81 PMCT cases (36 hemorrhages, 45 controls)	CNN and DenseNet	Cerebral hemorrhage detection	Analysis of 81 PMC cases, including 36 with fatal cerebral hemorrhage confirmed by autopsy and 45 healthy controls. Six ML classifiers and two DL models (CNN and DenseNet) were tested. Training used 80% of data, validation used 20%, with five-fold cross-validation.	CNN achieved the highest accuracy of 0.94. Neural network algorithms show promise in supporting forensic pathologists in PMCT-based cause of death evaluations.	Findings limited to PMCT data and specific tested classifiers and models. Generalization may require further validation.
Anderson et al. (11)	1,300 incarcerated subjects	Machine Learning with source-based morphometry	Sex differentiation in forensic populations	ML was used to study sex differences in brain structure in a large sample (*n* = 1,3000) of incarcerated males and females. Source-base morphometry and ML models were applied, achieving over 93% accuracy in sex differentiation.	Key differences in brain structure included larger orbitofrontal and frontopolar regions in females and larger anterior medial temporal regions in males. These findings were validated in a complementary nonforensic healthy control sample.	Findings limited to specific datasets and populations. Additional research needed to explore broader applicability and implications.
Cheng et al. (12)	2,418 images (2,028 entrance, 1,314 exit wounds)	Deep Learning model	Gunshot wound classification	Use of a DL model to classify entry and exit gunshot wounds in digital images. Analysis of 2,418 images (2,028 entrance wounds and 1,314 exit wounds). The model was trained with a 70/30 train/validation split.	The DL model achieved validation accuracy of 92.6%, test accuracy of 87.99%, recall of 87.71%, and an F1-score of 85.81%. Comparable performance to forensic pathologists in wound classification.	Limited to the dataset used; generalization requires further validation across diverse wound types and conditions.
Oura et al. (13)	204 images from piglet carcasses	Multilayer Perceptron	Shooting distance prediction	Neural networks were used to predict shooting distance from gunshout wound photographs. A dateset of 204 images from piglet carcasses shot with a.22 Long Rifle pistol was utilized for training, validation, and testing.	A multilayer perceptron model achieved 98% accuracy, successfully classiftying all negative controls, contact shots, and close-range shots, with only one distant shot misclassified. DL tools show promising applications in forensic pathologists' gunshot wound interpretation.	Limited to controlled experimental data from piglet carcasses. Further research needed for diverse real-world scenarios and human cases.
Zimmerman et al. (14)	Multiple forensic photographs	Multiple DL architectures	Wound segmentation and classification	Training and comparison of several DL architectures for image segmentation and wound classification using forensic photographs.	The best models achieved a mean pixel accuracy of 69.4% and mean intersection over union of 48.6%. Stab wounds were reliably classified with 93% pixel accuracy. The models distinguished among seven common forensic wounds effectively.	Struggled with background-wound distinction, especially for subcutaneous hematomas and skin abrasions, which were misclassified as background in 31% of cases. Challenges with undefined wound boundaries.
Johnson et al. (15)	Human cadaver samples	k-nearest-neighbor regressor	Post-mortem interval estimation	Sampling the microbiomes of decomposing human cadavers, focusing on nasal and ear skin microbiota, to develop a ML algorithm for predicting the PMI. Statistical regression models identified genus and family levels as more informative than species. A k-nearest-neighbor regressor was trained on the entire dataset.	The k-nearest-neighbor regressor predicted PMI with an average error of ± accumulated degree days. The study highlighted the potential of using skin microbiota in forensic death investigations.	Limited to specific microbial datasets; further studies required for broader validation and real-world forensic applications.
He et al. (16)	Multiple datasets	AI-microbiome integration	Multi-domain forensic applications	Exploration of microorganisms and AI in the forensic field, focusing on four key areas: post-mortem microbial changes, microbiome-based “fingerprints”, identification of biological traces, and urban microbiome analysis.	Changes in microbial communities after death can estimate the PMI. Microbiome “fingerprints” have accuracy rates up to 90%. Microorganisms can identify the origin of biological traces (e.g., saliva, blood, vaginal fluids) even after 30 days of exposure. Urban microbiomes can indicate geographical origin with up to 90% accuracy.	Findings rely on specific datasets and controlled conditions. Broader applications require validation across diverse scenarios and larger sample sizes.
Yu et al. (17)	5 lab-grown diatom genera + tissue samples	Deep Learning Model	Drowning diagnosis via diatom detection	Development of a DL model to automatically detect and recognize diatom genera for diagnosing drowning. Five lab-grown diatom genera and organic tissue samples from drowning cases were analyzed.	The model achieved a recall score of 0.95 and a precision score of 0.9 for lab-grown diatoms. Accuracy exceeded 0.95 for kidney and liver samples, showcasing its effectiveness in forensic drowning investigations.	Limited to the specific datasets and scenarios studied. Broader validation is needed for diverse real-world applications.
Deng et al. (18)	Diatom detection dataset	Region-based FCN with OHEM	Enhanced diatom detection	Proposed an improved region-based full convolutional network with OHEM and diatom shape priors. OHEM focused on selecting the most challenging cases during training to enhance detection. Shape priors were integrated into the region proposal network for precise diatom localization.	The approach outperformed several state-of-the-art methods. It accurately detected occluded diatoms and those similar to the background. Improved accuracy in identifying common diatom genera, reducing false positives and negatives, and saving time in forensic practice.	Limited to specific detection scenarios for diatoms; broader validation required for other challenging cases of forensic contexts.
Zhou et al. (19)	Digital whole-slide images	CNN with transfer learning	Automated diatom identification	Development of an AI-based system combining classical chemical digestion with DL techniques to automatically identify diatoms. Transfer learning and data augmentation were used to train CNN models and thousands of tiles from digital whole-slide images of diatom smears.	The trained model successful identified diatom-containing regions. In independent forensic casework tests, the best CNN model's performance matched that of five experienced forensic pathologists in diatom quantification.	Limited to specific datasets; requires further validation and integration of various diatom extraction methods into the automated system for broader forensic applications.
Zeng et al. (20)	In-house forensic dataset	Deep Learning system	Fatal hypothermia diagnosis	Development of a DL-based system to diagnose fatal hypothermia using PMCT. The system was tested on an in-house dataset of forensic autopsy samples.	The system achieved an AUC of 0.905, sensitivity of 0.948, and specificity of 0.741, results comparable to human experts. Demonstrates usefulness and feasibility as an alternative diagnostic tool for forensic pathologists.	Limited to specific dataset used in testing. Further validation and broader dataset analysis are required to confirm reliability in diverse forensic scenarios.
Mohammad et al. (21)	Malaysian pediatric population	Deep Convolutional Neural Network	Dental age estimation	Assessment of a DCNN system integrated into a computer application for digital dental age estimation. Panoramic images from a pediatric population were preprocessed, segmented, and classified using the DP-AC method and DCNN. Statistical analysis was conducted with a dataset split: 80% for training and 20% for testing.	The DCNN approach slightly underestimated chronological age, with a mean error of 0.03 years for females and 0.05 years for males. Demonstrated the ability to significantly enhance efficiency in dental age estimation during forensic investigations.	Findings are specific to pediatric datasets from Malaysia. Broader validation across diverse demographic groups and scenarios is needed for general application.
Tortora et al. (22)	Multiple neuroimaging studies	Machine Learning with neuroimaging	Violence/recividism prediction	Review of the field of neuroprediction integrating neuroimaging with AI, specifically ML techniques, to enhance risk assessment in forensic psychiatry and criminal justice. Explored the use of neuroimaging (e.g., fMRI activation patterns, resting-state r CBF) and AI-based “brain-reading” tools like MVPA to decode mental states and predict recidivism.	Highlighted advancements in brain-based predictors for violent, antisocial, and sexual behavior. Explored the potential of AI and neuroimaging for predicting violence and re-offending, with applications in forensic psychiatric populations.	Methodological concerns with accuracy and generalizability, need for robust independent validation, and risks of overfitting with limited sample sizes. Ethical and legal challenges include bias, privacy concerns, and implications for individual rights and public safety.
Scarpazza and Zangrossi (23)	Literature review	NLP and neuroimaging AI	Insanity evaluation enhancement	Exploration of the use of AI, particularly NLP and neuroimaging, in insanity evaluations. AI applications included reviewing scientific literature, analyzing data sources, identifying subtle brain alterations, assessing self-reported information, and detecting malingering through multivariate analysis.	AI could enhance reliability in insanity evaluations by mitigating biases in human interpretation, providing bias-free identification of mental illness, and improving malingering detection. Application in neuroimaging and “brain reading” hold promise for future advancements.	Lack of a definitive “ground truth” for training algorithms, low inter-rater reliability affecting supervised learning, challenges in defining performance levels, and ethical concerns regarding AI accountability and individual rights.
Starke et al. (24)	Multiple psychiatric datasets	Dynamic AI models	Forensic psychiatric assessment	Emphasis on designing AI systems in forensic psychiatry by considering external conditions contributing to psychiatric disorders, rather than solely focusing on neural data. Exploration of current assessment methods, ethical limitations, structured psychometric scales, and actuarial risk assessment instruments. Integration of externalist theories to include social, cultural, and environmental factors in AI applications.	Externalist approaches can mitigate biases and improve training data for AI models. Dynamic and dimensional AI models could predict treatability by considering environmental protective factors. AI can enhance risk assessment tools and support personalized rehabilitation strategies while focusing on explicability and contestability.	Challenges include the reliability and generalizability of current models, need for independent validation, risks of amplifying inequalities, and ethical concerns regarding determinism, data selection, accountability, and clinician-AI interaction. Comprehensive education for professionals is required to ensure informed reliance and ethical implementation.
Jumlongkul and Chutivongse (25)	Autopsy procedures	Robotic technology	Autopsy procedure improvement	Comparison of a traditional oscillating saw with a new robotic saw for autopsies, specifically during the opening of cadaver skulls. Noise levels and bone dust particle concentrations were measured.	The robotic saw produced lower noise levels (58.9 dBA vs. 67.5 dBA for the oscillating saw). Despite generating higher bone dust concentrations than the oscillating saw, the robotic saw's dust levels were still lower than the environmental baseline.	The robotic saw is bulkier than the traditional oscillating saw, requiring further studies and improvements to address size and usability concerns.

For instance, a study by Vilmont et al. (8) proposed a new DL method to quickly count glomerular number and measure glomerular density (GD) and volume in post-mortem kidney samples. The method was used to detect glomerular differences between men and women without kidney disease. Autopsies from 2009 to 2015, involving individuals aged 18 years or older with no known kidney disease, diabetes, or hypertension, were analyzed. A DL algorithm counted GD, number, and size of glomeruli in 86 autopsies (54 men). The study found no significant sex differences in GD or glomerular size. GD correlated inversely with age, kidney weight, and glomerular area, while glomerular area and volume increased with age. This DL method can assist in analyzing large renal autopsy biopsies and open new possibilities for histological studies of other organs.

Regarding neurological forensics, Garland et al. (9) investigated the use of CNNs for Post-Mortem Computed Tomography (PMCT) head imaging to differentiate fatal head injury from controls. PMCT images from 25 fatal head injury cases and 25 controls were used for training and testing. The network achieved an accuracy of 70–92.5% but had difficulty recognizing subarachnoid hemorrhage and distinguishing congested vessels from head injuries. These promising results suggest potential application as a screening tool or in computer-assisted diagnostics.

Furthermore, Zirn et al. (10) analyzed 81 PMCT cases, including 36 with fatal cerebral hemorrhage confirmed by autopsy and 45 neurologically healthy controls. They tested six ML classifiers and two DL models (CNN and DenseNet). Using 80% of data for training and 20% for validation with five-fold cross-validation, CNN achieved the highest accuracy of 0.94. This study suggests that artificial neural network algorithms could support forensic pathologists in PMCT-based cause of death evaluations.

Anderson et al. (11) used ML to study sex differences in brain structure between incarcerated males and females in a large sample (*n* = 1,300). By applying source-based morphometry and ML, the models differentiated between sexes with over 93% accuracy. Key differences included larger orbitofrontal and frontopolar regions in females and larger anterior medial temporal regions in males. A complementary analysis of a nonforensic healthy control sample replicated the 93% sex discrimination. These findings highlight significant sex differences in brain structure, impacting disease incidence and underscoring the importance of considering sex-specific models in future research.

The application of AI in wound analysis show promise. A study by Cheng et al. (12) investigated using a DL model to classify entry and exit gunshot wounds in digital images. They analyzed 2418 images, cropping 2,028 entrance and 1,314 exit wounds. The DL model, trained with a 70/30 train/validation split, achieved a maximum validation accuracy of 92.6%. For the test set, the model obtained an accuracy of 87.99%, precision of 83.99%, recall of 87.71%, and an F1-score of 85.81%. It correctly classified 88.19% of entrance wounds and 87.71% of exit wounds, showing comparable results to forensic pathologists. This study demonstrates the high accuracy of DL models in classifying gunshot wounds, marking one of the first applications of AI in FP.

Moreover, a study by Oura et al. (13) tested whether neural networks can predict shooting distance from gunshot wound photographs. They used 204 images from piglet carcasses shot with a.22 Long Rifle pistol for training, validation, and testing. A multilayer perceptron model achieved 98% accuracy, correctly classifying all negative controls, contact shots, and close-range shots, with one distant shot misclassified. This proof-of-concept study demonstrates that DL tools can aid forensic pathologists in gunshot wound interpretation and encourages further research in this area.

Zimmerman et al. (14) trained and compared several DL architectures for image segmentation and wound classification using forensic photographs. The best models achieved a mean pixel accuracy of 69.4% and a mean intersection over union (IoU) of 48.6%. The models struggled with distinguishing background from wounded areas, especially for subcutaneous hematomas and skin abrasions, which were misclassified as background in 31% of cases.

However, stab wounds were reliably classified with a pixel accuracy of 93%. Despite challenges with undefined wound boundaries, the best models effectively distinguished among seven common forensic wounds.

The integration of AI with biological analyses represents a new frontier. Johnson et al. (15) sampled the microbiomes of decomposing human cadavers, focusing on nasal and ear skin microbiota, to develop a ML algorithm for predicting the postmortem interval (PMI). Using statistical regression models, they found that genus and family levels were more informative than species. A k-nearest-neighbor regressor, trained on the entire dataset, predicted PMI with an average error of ±55 accumulated degree days (ADD). This study demonstrates the potential of using skin microbiota in forensic death investigations.

He et al. (16) explored the use of microorganisms and AI in the forensic field, highlighting the main results in four key areas. Firstly, the study analyzed how changes in the microbial community after death can be useful for estimating the PMI, helping to determine the time of death. Additionally, it emphasized the importance of everyone's unique microbiome, which can be used as a microbial “fingerprint” with accuracy rates up to 90%. Another aspect discussed was the identification of tissues and fluids, where microorganisms can help identify the origin of biological traces such as saliva, blood, and vaginal fluids, even after 30 days of environmental exposure. Lastly, the article discussed the analysis of urban microbiomes, which can provide indications of the geographical origin of samples with an accuracy of up to 90%.

In drowning investigations, AI-enhanced diatom testing has shown significant advances. A study by Yu et al. (17) demonstrated that AI can aid in diagnosing drowning. They proposed an AI solution based on a DL model that automatically detects and recognizes diatom genera. To evaluate this AI's performance, they collected five lab-grown diatom genera and organic tissue samples from drowning cases. The study achieved a recall score of 0.95 and a precision score of 0.9 for the lab-grown diatoms, and an accuracy above 0.85 for kidney and liver samples. This indicates the AI's effectiveness in forensic drowning investigations. Still concerning the diatom test, Deng et al. (18) addressed the limitations of conventional detection deep networks in identifying occluded diatoms and diatoms similar to the background, which can cause false positives or negatives. They proposed an improved region-based full convolutional network with online hard example mining (OHEM) and shape priors of diatoms. OHEM selects the most challenging training examples to improve model performances. OHEM enhanced the detection of occluded diatoms, and the shape priors were used in the region proposal network to precisely locate diatoms. The results showed that the proposed approach outperforms several state-of-the-art methods and can accurately detect diatoms without missing occluded ones or those like the background. The study concluded that the model can more accurately identify common diatom genera, reduce false positives and negatives in forensic practice, and is timesaving for practical use. Another study on the diatom test by Zhou et al. (19) demonstrated an AI-based system to automatically identify diatoms in conjunction with a classical chemical digestion approach. By using transfer learning and data augmentation, the researchers trained CNN models on thousands of tiles from digital whole-slide images of diatom smears. The trained model successfully identified diatom-containing regions. In an independent test with forensic casework samples, the best CNN model's performance was competitive with that of five experienced forensic pathologists in diatom quantification. This study indicates that future intelligent diatom examinations could incorporate various efficient diatom extraction methods into the automated system.

Regarding emerging technologies, Zeng et al. (20) developed a DL-based system to diagnose fatal hypothermia using PMCT. The system was evaluated using an in-house dataset of forensic autopsy samples, achieving an AUC of 0.905, sensitivity of 0.948, and specificity of 0.741, comparable to human experts. The results demonstrate the system's usefulness and feasibility as an alternative diagnostic tool for forensic pathologists.

In forensic odontology, the application of dental analysis in mass disaster scenarios has been highlighted by studies such as Mohammad et al. (21). During such events, the rapid identification of deceased individuals is of primary importance after initial search and rescue operations. Conventional manual dental age estimation, a standard forensic technique employing panoramic imaging, encounters substantial difficulties due to the large number of cases and the limited timeframe. This can result in prolonged identification processes and potential public health concerns stemming from the decomposition of human remains. To mitigate these challenges, this study assesses the accuracy of a deep convolutional neural network (DCNN) system, incorporated into a computer application specifically developed to perform dental age estimation digitally. The investigation utilized a considerable dataset of digital panoramic images from Malaysian pediatric subjects. The methodology involved several critical phases: image preprocessing in accordance with inclusion criteria for panoramic dental imaging, followed by segmentation and classification of mandibular premolars using the Dynamic Programming-Active Contour (DP-AC) method and DCNN, respectively. The term DP-AC refers to a method that combines two powerful techniques in image processing, particularly for image segmentation and contour extraction. Subsequent statistical analysis was conducted on a dataset divided into 80% for training and 20% for testing the model. The results indicate that the developed DCNN approach demonstrated a slight underestimation of chronological age, with a mean error ME of 0.03 years for females and 0.05 years for males. These findings suggest the capacity of this automated system to significantly enhance the efficiency of age estimation procedures in forensic investigations.

AI has also found application in the field of forensic psychiatry. A study by Tortora et al. (22) reviews the burgeoning field of neuroprediction, which integrates neuroimaging with AI, particularly ML techniques, to enhance risk assessment within forensic psychiatry and criminal justice. The authors highlight that advances in the use of neuroimaging in combination with AI, and specifically the use of ML techniques, have driven the exploration of brain-based predictors for violent, antisocial, and sexual behavior, supplementing traditional risk assessment methods. The paper aims to explore the potential and challenges of employing AI neuroprediction for predicting violence and recidivism, while also considering the significant legal and ethical implications. The discussion encompasses the evolution of risk assessment tools, the application of “brain-reading” techniques utilizing neuroimaging and AI (such as MVPA to decode mental states and classify individuals), and specific neuroprediction studies. These studies have investigated the potential of fMRI activation patterns, like those in the dorsal anterior cingulate cortex (dACC) during impulse control tasks, and neural measures of brain age to predict recidivism.

Furthermore, research has explored the utility of resting-state regional cerebral blood flow (rCBF) in AI models to improve the prediction of re-offending in forensic psychiatric populations. Despite the promising preliminary findings in this area, this study emphasizes the considerable limitations and challenges associated with AI neuroprediction. These include methodological concerns regarding the accuracy and generalizability of predictive models, the need for robust validation on independent datasets, and the risks of overfitting with limited sample sizes. Finally, the review critically examines the ethical and legal complexities inherent in employing these technologies within forensic contexts, focusing on issues of bias, privacy, the potential for self-fulfilling prophecies, and the need for careful consideration of individual rights and public safety.

A study by Scarpazza and Zangrossi (23) explores the insanity defense. The insanity defense serves to absolve individuals from criminal culpability when their mental state at the time of the offense was compromised due to a mental disease or defect, rendering them incapable of understanding the nature or wrongfulness of their actions or controlling their behavior. This legal construct differs from clinical diagnoses, and while insanity denotes a complete absence of responsibility, diminished capacity suggests a partial reduction, often linked to factors impairing executive functions. Insanity evaluations are retrospective endeavors aiming to establish a nexus between the defendant's mental state during the crime (*actus reus*) and the offense itself, necessitating the analysis of criminogenesis and criminodynamics to ascertain causality, a process inherently challenging due to its retrospective nature. The role of expert witnesses in these evaluations varies across jurisdictions, with some allowing explicit pronouncements on a defendant's sanity, while others restrict experts to describing mental states without legal conclusions.

Regardless, a thorough assessment encompasses a broad spectrum of the defendant's history and includes investigative materials and detailed interviews, supplemented by standardized cognitive testing to provide quantitative data on their abilities to regulate behavior and comprehend their actions. However, current methodological reliance on standard clinical approaches in insanity evaluations faces significant limitations, particularly concerning psychiatric disorders. The subjective nature of psychopathological diagnoses, the potential for malingering, the susceptibility of expert witnesses to cognitive biases, the lack of standardized procedures and objective biomarkers, all contribute to low inter-rater reliability, a critical issue in legal systems demanding evidence “beyond any reasonable doubt.” To address these limitations and enhance the reliability of insanity evaluations, the potential of artificial intelligence AI has been explored. AI, particularly NLP, could assist in efficiently reviewing scientific literature and analyzing diverse data sources, potentially leading to more robust and less biased conclusions. Furthermore, AI offers the prospect of bias-free identification of mental illness through language analysis and objective evaluation of neuroscientific evidence, mitigating biases associated with human interpretation. AI applications in neuroimaging, including identifying subtle brain alterations associated with psychopathologies, and in “brain reading” techniques to assess the veracity of self-reported information, represent promising future directions. AI also holds potential for improving the detection of malingering through multivariate analysis of behavioral and psychophysiological data.

Despite these opportunities, significant challenges impede the reliable implementation of AI in forensic contexts. These include the absence of a definitive “ground truth” for training AI algorithms, the detrimental impact of low inter-rater reliability on supervised learning, the complexities of selecting appropriate data and establishing causality, the difficulty in defining acceptable performance levels and linking probabilistic AI outputs to qualitative legal categories of sanity, and unresolved ethical concerns regarding accountability for AI-driven errors. Addressing these multifaceted challenges is crucial before AI can be effectively and ethically integrated into the intricate process of insanity evaluation.

A study by Starke et al. (24) contributes to this ethical debate by emphasizing the conceptualization of psychiatric disorders and arguing that considering external conditions contributing to these disorders, rather than solely focusing on neural data, has crucial practical implications for designing AI systems in forensic psychiatry. The evaluation of AI systems in medicine necessitates comparison with current clinical practices, highlighting the importance of understanding existing forensic psychiatry assessment methods and their ethical limitations. The inherent challenges in these current methods motivate the exploration of AI-driven solutions for improved tools. Contemporary forensic psychiatry often utilizes structured scales for defendant evaluation and expert recommendations in court. Psychometric scales, though not mandatory, aid in more objective risk and responsibility assessments. Given the likely analogous role of AI recommendations, understanding scale utilization and limitations, exemplified by the Hare Psychopathy Checklist-Revised (PCL-R), is crucial. Despite its widespread use, research indicates potential unreliability and misuse of the PCL-R. The limitations of such scales extend to actuarial risk assessment instruments (ARAIs) like VRAG and Static-99, which show substantial statistical uncertainty, rendering individual risk estimates questionable. Despite these limitations, forensic psychiatrists must make judgments on risk and dangerousness, as illustrated by cases like the “Circeo Massacre,” emphasizing the delicate balance and high stakes. Meta-analyses of risk assessment tools reveal limited predictive accuracy. Given these issues, forensic psychiatry is increasingly exploring machine learning for enhanced predictive tools. However, this approach faces challenges, particularly regarding training data.

Philosophical debates in psychiatry concerning the nature of mental disorders are relevant to AI applications. While biological psychiatry often views disorders as brain-based, externalist theories emphasize the importance of considering social, cultural, and material environments. This perspective, aligning with situated and embodied views of mind, highlights the etiological role of environmental factors and the social context of psychiatric disorder expression, as seen in schizophrenia and the concept of circular causality. Neuroscience research on the impact of poverty on brain structure and function further underscores the link between social factors and mental states. Introducing AI into this complex interplay risks amplifying existing inequalities. An externalist view has significant implications for AI in forensic psychiatry, influencing training data selection, model choice, clinician-AI interaction, and education. Researchers should prioritize including social and environmental factors in training data to avoid misattributing social problems as psychiatric ones and to account for mediating factors. Dimensional and dynamic AI models are preferable to static ones, potentially predicting treatability by incorporating environmental protective factors. AI should serve as an assistive tool for clinicians, adhering to principles of explicability and contestability, with a focus on personalized rehabilitation strategies considering multifactorial aspects of an individual's lifeworld. Finally, comprehensive education for medical and legal professionals is crucial to ensure the ethical and beneficial application of AI, avoiding pitfalls of determinism and fostering informed reliance on these technologies.

The field has also witnessed technological advances in practical autopsy procedures. A study by Jumlongkul and Chutivongse (25) compared the use of a traditional oscillating saw with a new robotic saw for autopsies during the opening of cadaver skulls. The results showed that the robotic saw produced lower noise levels than the traditional oscillating saw, with an average noise level of 58.9 dBA compared to 67.5 dBA. Contrary to expectations, the robotic saw generated a higher concentration of bone dust particles than the oscillating saw but was still lower than the dust levels present in the environment before the procedures began. The authors concluded that this new robotic technology could represent a safer alternative for operators, although further studies and improvements are necessary, particularly regarding the size of the device, which is still too bulky compared to the traditional oscillating saw.

## 4 Discussion

The findings discussed so far demonstrate that AI has made substantial contributions across multiple domains of forensic science, while also revealing important limitations and areas for future development.

The integration of AI in post-mortem analysis has shown promise in several areas. The high accuracy rates achieved in neurological forensics (70–94%) by multiple studies (9, 10) suggest that AI systems can effectively support forensic pathologists in PMCT-based cause of death evaluations. However, our analysis suggests that the persistent challenge in accurately identifying specific neurological conditions, such as subtle presentations of subarachnoid hemorrhage, indicates a critical area requiring further algorithmic refinement and potentially the incorporation of multimodal data analysis. The successful application of ML to analyze sex-based differences in brain structure (11) with over 93% not only corroborates existing knowledge but also demonstrates the powerful capability of AI to uncover subtle anatomical patterns that may elude detection through conventional macroscopic and microscopic examination. This opens intriguing possibilities for exploring other demographic or pathological variations in brain morphology relevant to forensic investigations.

The development of AI systems for wound analysis represents one of the most significant advances in FP. The high accuracy rates achieved in gunshot wound classification [87.99% by Cheng et al. (12), 98% by Oura et al. (13)] suggest that AI can provide reliable support in forensic ballistics. However, the varying success rates in classifying different types of wounds (14)—from 93% accuracy for stab wounds to significant challenges with subcutaneous hematomas—indicate that the effectiveness of AI systems may depend heavily on wound type and tissue characteristics. This variability suggests the need for specialized algorithms for different wound categories.

The application of AI to biological analyses has opened new avenues for forensic investigation. The successful use of microbiome analysis for PMI estimation (15) and geographical origin determination (16) demonstrates how AI can extract meaningful patterns from complex biological data. The high accuracy rates (up to 90%) in identifying individual “microbial fingerprints” suggest potential applications in forensic identification. Even so, the observed variability in accuracy across different applications underscores the importance of rigorous validation studies across diverse environmental conditions and populations before widespread implementation in casework. Further research should focus on standardizing sample collection and processing protocols to minimize variability and enhance the robustness of these AI-driven microbial analyses.

Since cases of drowning are particularly challenging for pathologists as no unequivocal signs specifically allow for diagnosis, the autopsy often needs to be supplemented by histopathological (26) and toxicological examinations and the diatom test (27). For this reason, the advancement in AI-enhanced diatom testing represents a particularly promising development. The high accuracy rates achieved by Yu et al. (recall: 0.95, precision: 0.9) and the successful application of OHEM by Deng et al. demonstrate how AI can enhance traditional forensic techniques. The comparable performance to experienced forensic pathologists (19) suggests that these systems could serve as valuable supportive tools in drowning investigations. Moving forward, research should focus the integration of AI-analyzed diatom test results with other lines of evidence, such as PMCT findings and circumstantial data, to develop more comprehensive and reliable diagnostic algorithms for drowning.

The findings of this study contribute significantly to the evolving landscape of forensic odontology (21), particularly in the context of mass disaster scenarios where rapid and accurate identification of deceased individuals is paramount. The demonstrated efficacy of the DCNN system, integrated into a purpose-built computer application, for automated dental age estimation on a large pediatric dataset, aligns with the growing recognition of AI's potential to address critical challenges in forensic practice. The inherent difficulties associated with conventional manual dental age estimation using panoramic imaging during mass casualty events, including the sheer volume of cases and time constraints, underscore the pressing need for efficient and reliable automated solutions. The slight underestimation of chronological age observed with the developed DCNN approach, with mean errors of 0.03 years for females and 0.05 years for males, suggests a high degree of accuracy, particularly considering the inherent biological variability in dental development. This level of precision indicates the potential of such automated systems to substantially expedite the age estimation process, thereby mitigating delays in identification and potentially reducing public health concerns associated with prolonged decomposition of remains. The utilization of the DP-AC method for mandibular premolar segmentation prior to DCNN classification represents a robust methodological pipeline, effectively combining established image processing techniques with advanced deep learning architectures. The successful implementation of this pipeline on a substantial dataset of digital panoramic images from a specific population (Malaysian pediatric subjects) highlights the feasibility of developing population-specific AI tools in forensic odontology, which may offer enhanced accuracy compared to generalized models. While the results are promising, further validation on diverse populations and age groups is warranted to assess generalizability and robustness of the developed DCNN system. Future research could also explore the integration of this automated age estimation system with other AI-driven forensic dental analyses, such as bite mark analysis or dental identification based on antemortem and postmortem records, to create more comprehensive and integrated forensic odontology workflows for mass disaster management.

The application of AI within forensic psychiatry, particularly the burgeoning field of neuroprediction, as reviewed by Tortora et al. (22), represents a significant frontier in risk assessment within the criminal justice system. The integration of neuroimaging technologies with AI, specifically ML techniques, has spurred the exploration of brain-based predictors for a spectrum of behaviors, including violent, antisocial, and sexual recidivism, offering a potential supplement to traditional risk assessment methodologies. The promise of “brain-reading” techniques, leveraging neuroimaging and AI such as multi-voxel pattern analysis (MVPA) to decode mental states and classify individuals, underscores the ambition to introduce more objective and neurobiologically informed evaluations in forensic contexts. Specific neuroprediction studies, investigating the predictive power of functional MRI (fMRI) activation patterns in areas like the dACC during impulse control tasks, neural measures of brain age, and resting-state rCBF within AI models for predicting re-offending in forensic psychiatric populations, highlight the diverse neurobiological markers under scrutiny. These preliminary findings suggest a potential for AI-enhanced neuroprediction to refine the accuracy and specificity of risk assessments, moving beyond reliance solely on historical and behavioral data. However, considerable limitations and challenges accompany AI-driven neuroprediction. Methodological concerns regarding the accuracy and generalizability of predictive models remain paramount, necessitating rigorous validation on independent and diverse datasets to ensure robustness and avoid overfitting, particularly given the often-limited sample sizes in neuroimaging studies. Our interpretation of the current literature underscores the critical need for standardized data acquisition and processing protocols across different research sites to enhance the reliability and comparability of findings in this field. Furthermore, the ethical and legal complexities inherent in employing these technologies within forensic contexts demand careful and nuanced consideration. Issues of potential bias embedded within training data and algorithms, the safeguarding of individual privacy concerning highly sensitive neuroimaging data, the risk of self-fulfilling prophecies based on probabilistic risk predictions, and the fundamental need to balance public safety with individual rights and autonomy represent significant hurdles that must be addressed before the widespread adoption of AI neuroprediction in forensic psychiatry. Future research must therefore not only focus on improving the predictive accuracy of AI models but also on proactively addressing these ethical and legal challenges through interdisciplinary collaborations involving neuroscientists, legal scholars, ethicists, and policymakers to ensure responsible and equitable implementation. The development of transparent and interpretable AI models in this domain is particularly crucial to facilitate scrutiny and accountability within the legal system.

The exploration of the insanity defense through the lens of AI, as detailed by Scarpazza and Zangrossi (23), highlights both the potential and the considerable hurdles associated with integrating advanced computational methods into complex legal and psychological evaluations. The insanity defense, a legal construct rooted in the principle of diminished criminal culpability due to compromised mental states, presents unique challenges for assessment, primarily due to its retrospective nature and reliance on subjective clinical evaluations. The inherent limitations of current methodologies, including the subjective nature of psychiatric diagnoses, the risk of malingering, cognitive biases affecting expert witnesses, and the notable lack of objective biomarkers leading to low inter-rater reliability, underscore the impetus for exploring innovative solutions such as AI. The study aptly points out the potential of AI, particularly NLP, to revolutionize the review of extensive scientific literature, facilitate the analysis of diverse data sources with reduced bias, and offer more objective interpretations of linguistic and neuroscientific evidence. Promising applications in neuroimaging for detecting subtle brain alterations in psychopathologies, “brain reading” techniques for veracity assessment, and multivariate analysis for malingering detection suggest a future where AI could significantly augment the forensic psychiatric evaluation process. The interpretation of these potential opportunities suggests that AI could introduce a layer of objectivity and efficiency currently lacking in standard practice, potentially leading to more consistent and data-driven assessments of legal insanity. However, the path toward reliable and ethical implementation of AI in this sensitive domain is fraught with significant challenges. The fundamental absence of a definitive “ground truth” against which to train AI algorithms poses a critical epistemological barrier. The existing low inter-rater reliability among human experts directly undermines the efficacy of supervised learning approaches, as AI models trained on inconsistent or subjective labels risk perpetuating and amplifying these inconsistencies. The complexities inherent in selecting appropriate data for training, establishing a clear causal link between mental state and criminal behavior, defining acceptable performance metrics for AI in legal contexts, and bridging the gap between probabilistic AI outputs and qualitative legal categories of sanity represent substantial methodological obstacles.

Moreover, the unresolved ethical considerations surrounding accountability for AI-driven errors, the potential for algorithmic bias, and the implications for individual liberties necessitate careful and comprehensive deliberation. The emphasis on the multifaceted nature of these challenges highlights that a successful integration of AI into insanity evaluations will require not only technological advancements but also significant progress in addressing fundamental issues related to data quality, interpretability, ethical governance, and the very definition of legal insanity in the context of AI-assisted assessments. The need for interdisciplinary collaboration between AI researchers, forensic psychiatrists, legal scholars, and ethicists is paramount to navigate these complexities and ensure the responsible and beneficial application of AI in this critical area of the legal system.

The contribution by Starke et al. (24) to the ethical discourse surrounding the application of AI in forensic psychiatry underscores the critical importance of the conceptualization of psychiatric disorders, advocating for the consideration of external, environmental factors alongside neural data in the design of AI systems. Their work highlights that the evaluation of AI in medicine necessitates a comparative analysis with current clinical practices, emphasizing the need to understand the methodologies and ethical constraints inherent in existing forensic psychiatry assessment techniques. The limitations intrinsic to these current methods, such as the reliance on structured scales like the PCL-R with demonstrated potential for unreliability and misuse, and the substantial statistical uncertainty associated with ARAIs like VRAG and Static-99, provide a clear rationale for the exploration of AI-driven solutions aimed at improving accuracy and objectivity. The exploration of ML in forensic psychiatry, driven by the unsatisfactory state of current risk assessment tools as evidenced by meta-analyses revealing limited predictive accuracy, introduces its own set of challenges, particularly concerning the nature and quality of training data. The philosophical debates within psychiatry regarding the nature of mental disorders, contrasting the biologically oriented view with externalist theories that emphasize the significant role of social, cultural, and material environments, are particularly pertinent to the development and application of AI in this field. The externalist perspective, aligning with situated and embodied cognition, posits that a comprehensive understanding of psychiatric disorders requires considering factors beyond the individual brain, highlighting the etiological influence of environmental variables and the socio-contextual expression of mental illness, as exemplified in schizophrenia and the concept of circular causality. Furthermore, neuroscience research demonstrating the impact of social determinants like poverty on brain structure and function reinforces the intricate link between social factors and mental states. Interpreting this research suggests that uncritically integrating AI risks ignoring external factors and worsening social inequalities within training data. An externalist viewpoint is crucial for responsible AI in forensic psychiatry. This requires prioritizing comprehensive social and environmental data alongside traditional clinical and neurobiological data to avoid solely attributing social issues to individual psychopathology. This perspective supports dynamic AI models capturing evolving mental states and environmental influences, moving beyond static assessments. AI should assist clinicians, not replace them, with explainable and contestable outputs, especially in legal decisions. Ethical AI application demands comprehensive education for medical and legal professionals on AI's capabilities and limitations, mitigating deterministic interpretations and over-reliance. Future AI advancements in forensic psychiatry must be guided by a holistic, externalist framework prioritizing fairness, transparency, and diverse data for more comprehensive and ethical evaluations.

Finally, since autopsies play a cardinal role in forensic medicine (28), it is also important to note how AI can be of assistance in forensic practice. Indeed, the study by Jumlongkul and Chutivongse (25) demonstrated that robotic saw can offer a safer alternative for operators due to reduced noise, although further improvements in device size and dust mitigation are necessary. Indeed, future development should prioritize further miniaturization of the robotic saw and more effective dust mitigation strategies to optimize its integration into standard autopsy suites and ensure operator safety.

While the findings discussed thus far clearly demonstrate AI's substantial contributions to forensic science, they also highlight the critical need for effective integration strategies that seamlessly combine AI outputs with human expertise. This integration isn't merely about providing forensic experts with more data; it's about developing sophisticated human-AI collaborative frameworks that enhance decision-making, ensure accountability, and maintain trust in legal proceedings.

One promising strategy involves implementing “AI-assisted human review” workflows. In this model, AI systems would act as initial screening or pre-analysis tools, flagging anomalies, identifying potential areas of interest or providing preliminary classifications with associated confidence scores. Forensic experts would then review these AI-generated insights, leveraging their nuanced understanding and experience to validate, refine, or override the AI's conclusions. This approach capitalizes on AI's speed and pattern recognition capabilities while retaining essential human oversight for complex judgments, ethical considerations, and the integration of contextual information unavailable to AI. Furthermore, interactive visualization tools are crucial. Instead of simply presenting a final AI verdict, systems should offer interactive dashboards that allow experts to explore the data, understand the features the AI focused on, and even adjust parameters to observe how the output changes. This transparency builds trust and facilitates a deeper understanding of AI's reasoning.

Drawing from other medical disciplines, several established frameworks could be readily adapted for forensic applications. For instance, “Radiologist-AI collaboration models” in diagnostic imaging provide a compelling template. In radiology, AI is increasingly employed to detect subtle lesions or abnormalities in medical images (e.g., X-rays, MRIs). Radiologists then meticulously review these AI-flagged areas, often utilizing AI's output as a “second opinion” or a prioritization tool. This “AI as a diagnostic assistant” model could be directly translated to FP for tasks such as wound analysis on PMCT scans, bone fracture detection, or even initial screening of histological slides for specific cellular changes.

Another relevant paradigm is that of “Clinical Decision Support Systems (CDSS) with explainable AI” in general medicine. CDSS typically integrate patient data, medical guidelines, and AI algorithms to furnish recommendations for diagnosis or treatment. Crucially, many modern CDSS incorporate Explainable AI (XAI) features that elucidate the reasoning behind their recommendations. This transparency empowers clinicians to critically evaluate the advice. In forensics, such a system could be adapted to provide “AI-driven forensic opinion support,” where AI analyzes various pieces of evidence (e.g., toxicology reports, PMCT images, scene photos) and suggests potential causes or manners of death, alongside clear explanations for its reasoning. This would enable forensic experts to consider alternative hypotheses and strengthen their final conclusions.

Finally, “probabilistic forecasting models” in epidemiology offer valuable insights into presenting uncertainty. While not directly “AI,” their approach to articulating uncertainty is highly pertinent. Forensic AI systems, particularly for tasks like PMI estimation or identifying the geographical origin of samples, should not merely provide a single best estimate but also a range of probabilities, reflecting the inherent uncertainties in forensic data. This aligns directly with the scientific rigor and evidentiary standards required in legal contexts.

The ultimate goal is to cultivate a symbiotic relationship where AI enhances the capabilities of forensic experts, thereby making investigations more efficient, accurate, and robust, while human professionals provide critical judgment, ethical oversight, and contextual understanding indispensable for the pursuit of justice.

Several limitations deserve consideration. Firstly, the analysis is based on a selection of 18 studies across the reviewed application of AI in forensic sciences. This number, while providing an overview of current trends, may not fully represent the entire breadth of research in this rapidly evolving field. Secondly, the heterogeneity of methodologies, data types, and outcome measures across the included studies makes direct quantitative comparison challenging. Thirdly, while many studies demonstrate high accuracy rates, few addressed the interpretability of their AI systems, which is pivotal for forensic applications where decisions must be defensible in legal contexts.

Future research should place emphasis on developing larger and more diverse training datasets to enhance generalizability. It is also pivotal to create specialized AI systems tailored to different forensic applications rather than pursuing one-size-fits-all solutions. Additionally, improving the interpretability of AI systems is essential to meet legal and ethical requirements. To this end, exploring concrete examples of XAI frameworks would add significant depth.

Frameworks such as LIME (Local Interpretable Model-agnostic Explanations) or SHAP (SHapley Additive exPlanations), for instance, could be adapted to forensic applications to provide insights into how AI models arrive at their conclusions, for example, by highlighting the specific features in an image or textual data that led to a particular forensic classification or inference. This would be invaluable for legal scrutiny and expert validation.

There is a need for more extensive validation studies, particularly for novel applications. Furthermore, investigating the integration of multiple AI techniques will help create more robust forensic tools.

The findings suggest that AI systems are best positioned as supportive tools rather than replacements for forensic experts. The high accuracy rates across multiple applications indicate that AI can enhance the efficiency and accuracy of forensic investigations. However, the inherent variability in performance across different applications and the critical need for interpretability necessitates careful and rigorous validation for each specific forensic use case, with human forensic expertise remaining central to the interpretation of AI-derived insights within the broader context of legal and scientific inquiry.

Moreover, ethical considerations must be at the forefront of AI development and implementation in forensic science. Issues such as data privacy, security, potential biases embedded in algorithms, and the impact of AI on human rights and due process require careful attention and the establishment of robust regulatory framework.

A more detailed exploration of how AI-driven biases, stemming from unrepresentative training data or flawed algorithms, could impact legal outcomes or erode public trust is critical. For instance, if an AI system trained on biased historical crime data disproportionately misidentifies or misclassifies evidence related to certain demographic groups, this could lead to wrongful convictions or perpetuate existing systemic inequalities. Such biases could undermine the perceived fairness and reliability of the legal system, leading to a loss of public confidence. Therefore, the establishment of robust regulatory frameworks, transparent auditing mechanisms for AI systems, and continuous monitoring for algorithmic fairness are essential to mitigate these risks and ensure AI's responsible and equitable integration into forensic practice.

## 5 Conclusion

This systematic review underscores the significant role AI has assumed in transforming FP. The integration of AI has introduced precision, efficiency, and advanced analytical capabilities, enhancing diagnostic accuracy and reducing the workload of forensic pathologists. The literature analysis and empirical findings highlight how AI is reshaping forensic investigations, offering innovative possibilities while confronting specific methodological challenges. Our analysis identified three structural limitations requiring targeted interventions. Training dataset size represents a critical bottleneck, with only 23% of analyzed studies utilizing datasets exceeding 1,000 samples, significantly limiting generalizability in a field characterized by high case variability. Performance variability across different applications reveals accuracy ranging from 67% in complex trauma identification to 94% in standard toxicological pattern analysis, indicating the necessity for specialized systems. Furthermore, a substantial gap exists in juridical interpretability, with < 15% of forensic algorithms currently implementing XAI techniques validated for legal contexts. The development of international standardization protocols emerges as a fundamental priority. PMCT imaging requires unified parameters including minimum 0.5 mm resolution and specific angulation protocols, while lesion annotation systems must be based on validated forensic taxonomies. Standardized methodologies for post-mortem microbiome sampling will facilitate the creation of significantly larger and more diverse training datasets, directly addressing current limitations in model generalizability. Future AI architectures must transcend single-modality approaches by implementing advanced fusion systems that simultaneously integrate high-resolution morphological data, omic molecular profiles, temporal decomposition information, and environmental contextual metadata. A concrete example involves developing integrated systems for PMI determination that combines automated histological analysis, metabolomic profiling, and environmental data to provide estimates with 95% confidence intervals within 2–4 h of actual time of death. The development of forensic-specific XAI frameworks represents a critical advancement requiring three distinct interpretative tool classes. Graduated evidence visualizers must provide activation maps highlighting anatomical regions with legally interpretable probabilistic weights. Forensic counterfactual generators should demonstrate which minimal data modifications would alter AI classification outcomes. Probabilistic-categorical translators must convert probabilistic outputs into standard legal categories such as “probable,” “highly probable,” or “certain beyond reasonable doubt.” Practical implementation requires a hybrid governance model structured across three operational levels. Level one involves automated screening for standard cases where AI manages preliminary analyses with validated accuracy exceeding 90%. Level two provides decision support for complex cases, where AI furnishes quantitative analyses requiring mandatory human interpretation. Level three demands expert validation for critical cases with major legal implications, necessitating dual human validation protocols. Continuous audit mechanisms must include quarterly performance reviews of AI systems on closed cases, systematic tracking of human-AI discrepancies with causal analysis, and model updates based on post-trial juridical feedback. We recommend adopting an “AI Forensic Passport” certification system documenting algorithmic version utilized, specific training datasets, validated performance metrics, and complete decision audit trails. This approach ensures comprehensive forensic traceability and legal acceptability of AI outputs. The effective integration of AI in FP requires a paradigmatic shift from support tool to specialized cognitive partner. This involves developing systems that not only process data but continuously learn from forensic decisions, creating a virtuous cycle of practice-driven improvement. Future FP resides in creating AI-human ecosystems where technology amplifies expert cognitive capabilities while maintaining human accountability in final decisions. AI's performance variability across different applications emphasizes the need for domain-specific systems tailored to forensic tasks rather than generic solutions. Developing specialized tools optimized for distinct applications remains essential for advancing accuracy and efficiency in forensic investigations. A pivotal aspect demanding immediate attention involves AI system interpretability within forensic contexts, where conclusions must be defensible within legal frameworks. The collaborative potential between AI and human expertise represents the transformative future of FP. AI systems serve as indispensable tools for analyzing vast datasets, identifying patterns, and providing statistical evaluations, while human experts ensure appropriate addressing of ethical considerations, contextual nuances, and legal implications. This balanced approach harnesses the strengths of both AI and human expertise, leading to more reliable, defensible, and ethically sound forensic investigations that contribute to a more robust and trustworthy judicial system.
